# A Novel Coupled Reaction-Diffusion System for Explainable Gene Expression Profiling

**DOI:** 10.3390/s21062190

**Published:** 2021-03-21

**Authors:** Muhamed Wael Farouq, Wadii Boulila, Zain Hussain, Asrar Rashid, Moiz Shah, Sajid Hussain, Nathan Ng, Dominic Ng, Haris Hanif, Mohamad Guftar Shaikh, Aziz Sheikh, Amir Hussain

**Affiliations:** 1 Department of Statistics, Mathematics and Insurance, University of Ain Shams, Cairo 11566, Egypt; m.wael.farouq@gmail.com; 2 School of Computing, Edinburgh Napier University, Edinburgh EH11 4BN, UK; 3 RIADI Laboratory, National School of Computer Sciences, University of Manouba, Manouba 2010, Tunisia; wadii.boulila@riadi.rnu.tn; 4 IS Department, College of Computer Science and Engineering, Taibah University, Medina 42353, Saudi Arabia; 5 College of Medicine and Veterinary Medicine, University of Edinburgh, Edinburgh EH8 9YL, UK; zain.hussain@ed.ac.uk (Z.H.); nathannghuang@gmail.com (N.N.); aziz.sheikh@ed.ac.uk (A.S.); 6 Midland Doctors, Derby DE73 7HA, UK; asrar@midlanddoctors.org; 7 NHS Greater Glasgow and Clyde, Glasgow G12 0XH, UK; moiz95@gmail.com (M.S.); guftar.shaikh@ggc.scot.nhs.uk (M.G.S.); 8 Albany Gastroenterology Consultants, Albany, NY 12206, USA; sajid.hu@gmail.com; 9 Faculty of Biology, Medicine and Health, University of Manchester, Manchester M13 9PL, UK; dominic.ng@student.manchester.ac.uk (D.N.); haris.hanif90@myhunter.cuny.edu (H.H.)

**Keywords:** gene expression, diffusion equation, coupled reaction PDE, non-small cell lung cancer, explainable machine learning

## Abstract

Machine learning (ML)-based algorithms are playing an important role in cancer diagnosis and are increasingly being used to aid clinical decision-making. However, these commonly operate as ‘black boxes’ and it is unclear how decisions are derived. Recently, techniques have been applied to help us understand how specific ML models work and explain the rational for outputs. This study aims to determine why a given type of cancer has a certain phenotypic characteristic. Cancer results in cellular dysregulation and a thorough consideration of cancer regulators is required. This would increase our understanding of the nature of the disease and help discover more effective diagnostic, prognostic, and treatment methods for a variety of cancer types and stages. Our study proposes a novel explainable analysis of potential biomarkers denoting tumorigenesis in non-small cell lung cancer. A number of these biomarkers are known to appear following various treatment pathways. An enhanced analysis is enabled through a novel mathematical formulation for the regulators of mRNA, the regulators of ncRNA, and the coupled mRNA–ncRNA regulators. Temporal gene expression profiles are approximated in a two-dimensional spatial domain for the transition states before converging to the stationary state, using a system comprised of coupled-reaction partial differential equations. Simulation experiments demonstrate that the proposed mathematical gene-expression profile represents a best fit for the population abundance of these oncogenes. In future, our proposed solution can lead to the development of alternative interpretable approaches, through the application of ML models to discover unknown dynamics in gene regulatory systems.

## 1. Introduction

A gene is a sequence of nucleotides along a deoxyribonucleic acid (DNA) strand, which is stored in the cell nucleus. Most genes that code for the production of proteins regulate cellular and tissue function, and specify the order of amino acid assembly to create particular proteins [1,2].

Gene expression is the process by which the nucleotide sequence of a gene is used to synthesize a specialized type of single-stranded molecule, termed messenger ribonucleic acid (mRNA), which is used to direct protein synthesis. Gene regulation refers to the mechanisms that control gene expression, resulting in a gene being active or suppressed. These include mechanisms which modulate translation of mRNA, structural changes to genetic material, or proteins binding to DNA and regulating transcription.

Like DNA, RNA is constructed from micro-molecules, termed nucleotides (nt). RNA molecules performing a function are termed ‘functional RNA’, whereas residual RNAs are termed ‘non-functional RNA’. Non-coding RNAs (ncRNAs) are synthesized and transcribed from DNA and do not encode for a protein [1,2]; rather, their mechanisms are tethered to small ncRNAs, including small interfering RNA (siRNA) and microRNA (miRNA), which regulate the process of mRNA destabilization, or translation inhibition [3,4,5]. Therefore, unlike DNA studies, RNA analysis has the potential to provide a dynamic functional appreciation of biological systems.

To better understand cancer and discover more effective diagnostic, prognostic, and treatment approaches, a thorough consideration of cellular regulators is necessary. Cancer encompasses a group of diseases in which abnormal cell growth results initially in localized affects, leading to cellular dysfunction. Subsequent cellular cancer progression, with or without dissemination, leads to organ dysfunction with the risk of major systemic effects. A more in-depth consideration of this set of neoplastic conditions begins with the fundamentals outlined above, whereby genes play a pivotal role in protein synthesis and regulate tumorigenesis. Moreover, cells are governed by a cell-cycle influenced by age, hence the activity of certain genes—whether in translation, upregulation, or suppression—can alter and disrupt cellular age or function, resulting in otherwise normal cells undergoing oncological transformation. Modulating the temporal and spatial concentrations of mRNAs can uncover new opportunities for treating and improving oncological prognosis.

Machine learning (ML) models have been applied to many types of datasets, e.g., gene expression, bio-imaging, and clinical datasets, and have been shown to accurately predict and diagnose cancer [6,7,8,9,10,11,12]. However, because outputs from such approaches are often used to inform a clinician’s decision-making, it is imperative that they are able to understand the rationale behind a model’s decision. Therefore, the proposition is that improved interpretable ML modelling will inform clinicians and enhance their decision-making [13,14,15,16]. There is significant potential to further develop ML models based on gene expression profiling, which would advance oncology therapeutics and management. Gadgil et al. [17] analyzed DNA microarray hybridization by drawing on the reversible hybridization reaction and then preparing a diffusional transport of the newly labelled strands. They also presented a diffusion-reaction model representing the microarray assays of complementary DNA. They aimed to simulate the hybridization of different strands corresponding to various types of mRNAs, both common and rare, and based their experiments on geometrically realistic domains for labelled DNA concentrations.

Wang et al. [18] used supervised machine learning (ML) algorithms to map and classify cancers using microarray data, to find the minimum gene subsets required to reduce computational burden and ‘noise’, which irrelevant genes had previously produced in other algorithms. Their proposed method was based on two steps: (1) using a ‘feature importance’ ranking scheme to select essential genes; and (2) testing the classification capacity, made possible using all simple combinations of the essential genes identified. A comparison of this approach with more recent machine learning models such as deep, reinforcement, and adversarial machine learning networks is still required [19].

Jing et al. [20] introduced a hybridized approach by drawing on the profiles of gene expression and gene ontology. Gene regulatory networks were constructed using both overlapping clustering and reverse engineering methods. The approach was validated using a yeast-cell-cycle dataset which demonstrated its ability to construct gene-regulatory networks.

Cho and Levy [21] proposed a mathematical model to analyze drug-resistance in solid tumors. The reaction–diffusion-based model followed the dynamics of the tumor, and studied how the tumor’s changes under chemotherapy were impacted by spatial and phenotypic heterogeneity.

Zhang et al. [22] employed reaction–diffusion terms with Dirichlet boundary conditions to address state estimation for delayed genetic regulatory networks (DGRNs). Their work introduced a state observer that estimated the concentrations of both mRNAs and proteins using readily available techniques for measurement. New integral terms were introduced into a Lyapunov-Krasovskii function, and experiments were conducted based on two numerical examples to test theoretical results.

Song et al. [23] developed a state estimator for genetic regulatory networks using reaction–diffusion terms. Measurement costs were reduced by dividing the diffusion space into multiple regions. To ensure the estimated errors fell to zero, a new criterion based on the Lyapunov functional was proposed and combined with Wirtinger’s inequality, the reciprocally convex approach, and Halanay’s inequality. The applicability of this approach was demonstrated using experiments based on two simulation examples.

In this paper, we propose an alternative, coupled reaction-diffusion system-based approach for explainable gene expression profiling. Specifically, we approximate the temporal expression profile of genes in a two-dimensional spatial domain for the transition states, before progressing to a stationary state, using a novel combination of coupled-reaction partial differential equations. Our approach is intended to help analyze biological biomarkers that could denote tumorigenesis in non-small lung cancer, following various treatment solutions. Non-small cell cancer represents 80–85% of all lung cancer cases. For many of these patients, the preferred mainstay of treatment involves a combination of systemic, cytotoxic therapy, and immunotherapies. Technological advancements in gene expression profiling could not only help improve current targeted drug therapies, but also improve prognosis biomarkers, diagnosis, and prediction [24]. The proposed approach could potentially identify novel and improved cancer treatment. Previous studies [10,12] have attempted other analyses of gene expression profiles, primarily through the development of mathematical and ML-based models. However, the use of the diffusion equation to study gene expression profiling is not yet well explored, as demonstrated through a brief survey of the literature above.

The main contributions of this study are summarized as follows:

1. An innovative mathematical explanation of potential biomarkers denoting tumorigenesis in non-small cell lung cancer.
2. A detailed formulation of the regulators of gene expression of non-thermal plasma treatment of non-small cell lung cancer: regulators of mRNA, the regulators of ncRNA, and the coupled mRNA–ncRNA regulators.
3. An approximation of the temporal expression-profile of genes in a two-dimensional spatial domain using a system comprised of coupled-reaction partial differential equations.
4. Various simulation experiments conducted to validate the proposed mathematical gene-expression profile.

Our proposed solution aims to help researchers understand unknown dynamics in gene regulatory systems by providing a novel mathematical formulation of potential biomarkers denoting tumorigenesis in non-small cell lung cancer. Further research could lead to the development of alternative interpretable approaches, through future applications of ML models to discover unknown dynamics in gene regulatory systems and other challenging domains [25,26,27,28].

The rest of our paper is organized as follows. Section 2 establishes the fundamental concepts of the diffusion equation and Section 3 describes the proposed computational and qualitative analysis based on two-species diffusion equations. Section 4 details the results of our simulation and, finally, Section 5 concludes our study and outlines future research directions.

## 2. Diffusion Equation

In this section, we describe the diffusion equation used to approximate the temporal expression profile of genes.

The one-dimensional diffusion equation is written as follows [29,30,31,32,33,34]:

(1) $$\frac{\partial u}{\partial t}=D\frac{\partial^{2}u}{\partial x^{2}}\;\left(0\leq x\leq L\;\mathrm{and}\;t\geq 0\right)$$

where (*u*) is the molecular, mass, or heat concentration, (*x*) is the spatial domain, and *D* is the diffusion coefficient.

The one-dimensional diffusion equation conveys that the molecular concentration diffuses with the rate *D* in the one-dimensional spatial domain in both directions without any preference. The rate of diffusion depends on *D*, which denotes the molecular, mass, or heat diffusion coefficient. The diffusion coefficient increases when the diffusion process is faster. The diffusion equation states that the molecular concentration (*u*) at any point depends only on the *x*-coordinate of the point and the time (*t*) [31,35,36].

The fact that the diffusion equation is a first order in time conveys that the diffusion is unidirectional in time. At any point, the diffusion depends on the previous time while considering the initial condition at (*t* = 0). Alternatively, when the diffusion equation is a second order in space, this conveys that the diffusion is in a two-space direction without any preference [33,36]. In this case, two boundary conditions at (*x* = 0) and (*x* = *L*) are required [32,33]. Hence, to obtain a unique solution, one initial condition and two boundary conditions are needed.

Various types of boundary conditions at (*x* = 0) and (*x* = *L*) are possible; the Dirichlet boundary conditions for a one-dimensional diffusion equation are presented by the following equation [29,32]:

(2) $$u\left(0,t\right)=g_{1}\left(t\right)\;\mathrm{and}\;u\left(L,t\right)=g_{2}\left(t\right)$$

If $g_{1}\left(t\right)=0$ and $g_{2}\left(t\right)=0$, the boundary conditions are said to be Dirichlet homogenous boundary conditions, whereas if $g_{1}\left(t\right)\neq 0$ and $g_{2}\left(t\right)\neq 0$, the boundary conditions are said to be Dirichlet non-homogenous or inhomogeneous boundary conditions. A system composed of a diffusion partial differential equation (PDE), an initial condition, and Dirichlet boundary conditions is referred to as a Dirichlet initial boundary value problem for the diffusion equation [34]. The Neumann boundary conditions, as depicted by the following equation [34], are another type of boundary equation:

(3) $$\frac{\partial u}{\partial x}\left(0,t\right)=g_{1}\left(t\right)\;\mathrm{and}\;\frac{\partial u}{\partial x}\left(L,t\right)=g_{2}\left(t\right)$$

If $g_{1}\left(t\right)=0$ and $g_{2}\left(t\right)=0$, then the boundary conditions are said to be Neumann homogenous boundary conditions. However, if $g_{1}\left(t\right)\neq 0$ and $g_{2}\left(t\right)\neq 0$ then the boundary conditions are said to be Neumann non-homogenous or inhomogeneous boundary conditions. A system composed of a diffusion PDE, an initial condition, and Neumann boundary conditions is referred to as a Neumann initial boundary value problem for a diffusion equation [32,37].

The general form of the multi-dimensional diffusion equation is written as follows:

(4) $$\frac{\partial u}{\partial t}=D∆u$$

where 0≤x≤L, 0≤y≤K, …, 0≤n≤G, and t≥0, and Δ is referred to as the Laplace operator, defined as [30,31]:

(5) $$∆u=\left(\frac{\partial^{2}u}{\partial x^{2}}+\frac{\partial^{2}u}{\partial y^{2}}+\ldots +\frac{\partial^{2}u}{\partial n^{2}}\right)$$

The two-dimensional counterpart diffusion equation is written as [26]:

(6) $$\frac{\partial u}{\partial t}=D\left(\frac{\partial^{2}u}{\partial x^{2}}+\frac{\partial^{2}u}{\partial y^{2}}\right)$$

where 0≤x≤L, 0≤y≤K and t≥0.

It is clear that if the homogenous diffusion equation is steady, then it is time independent and $\frac{\partial u}{\partial t}=0$. This implies the preceding equation can be written as follows:

(7) $$\frac{\partial^{2}u}{\partial x^{2}}+\frac{\partial^{2}u}{\partial y^{2}}=0$$

The preceding equation is a canonical form of the elliptic equation and is referred to as the Laplace equation, which indicates the steady state or equilibrium form of the diffusion equation [31,32]. Moreover, the non-homogenous diffusion equation without a time derivative is equivalent to the non-homogeneous form of the Laplace equation, which is referred to as the Poisson equation [31,32]. This is presented as follows:

(8) $$\frac{\partial^{2}u}{\partial x^{2}}+\frac{\partial^{2}u}{\partial y^{2}}=g\left(x,y\right)$$

For any second-order linear PDE, the diffusion equation is characterized by several properties, such as the maximum principle, the uniqueness of solutions, the invariance properties of the heat equation, and the stability of the solution [31,32,35,36,38,39].

## 3. Proposed Computational and Qualitative Analysis: Two-Species Diffusion Equation

In this section, the formulation of our proposed mathematical model for the mRNA–ncRNA interactions is introduced, based on the conceptual mathematical aspects discussed previously. We start with a model for the mRNA–ncRNA interactions within the cell considering these parameters: production rates, self-degradation death rates, dependent-coupled degradation-death rates, and delay rate for the three species of RNA molecules. Then, we detail the model for the three species interactions that fundamentally differ from the introductory model by introducing mRNA movement, ncRNA movement, and mutual species movement, between cells across the tissue.

Table 1 introduces notations to be discussed for our proposed mathematical model and its associated parameters.

The population of mRNA is given by Equation (9). The partial differential equation is based upon a two-species model thereby requiring differing inputs.

(9) $$m_{i}\left(x,y,t\right)=D_{m_{i}}\nabla m_{i}+f[\alpha_{m_{i}}x,y]-f[m_{i}(x,y,t)]-f[m_{i},ns]-f[m_{i},nm]$$

The population of siRNA is given by Equation (10).

(10) $$ns\left(x,y,t\right)=D_{ns}\nabla ns+f[\alpha_{ns}x,y]-f[ns]-f[m_{i},ns]$$

The system of partial differential equations penalizes the mRNA–ncRNA interactions that play a crucial role in gene regulation. We differentiate between the two non-coding RNA species in the gene regulation mechanism. Small interfering RNA (siRNA) regulates genes by causing degradation to the mRNA and hence is modelled using a system of coupled partial differential equations. However, micro-RNA regulates genes by inhibiting translation but eventually causes degradation. Thus, the gene regulation mechanism of the miRNA is modelled through a system of coupled-delay partial differential equations instead. Moreover, the molecular movement potency of mRNA and the two species of non-coding RNA within the tissue are both modelled by a diffusion paradigm.

Our numerical solutions and computational results show two species interactions in a two-dimensional environment. The two species are mRNA and nsRNA, and parameters for both (transcription rate, self-degradation rate, coupled degradation) were chosen based on experimental data and varied with newly introduced rates to show the sensitivity of parameter tuning to the mRNA population state. We note that population state does not refer to population abundance alone but also population structure.

We present population abundance and structure starting with a mixture of the Gaussian initial state, unless stated otherwise. We vary the two species model parameters to unveil different patterns for population states. We also present replicated results for each of the models with a variation to the initial state.

First: Coupled reaction ordinary differential equations (ODE)—Model for mRNA–ncRNA interactions within a cell:

(11) $$\frac{\partial m_{i}\left(t\right)}{\partial t}=\alpha_{m_{i}}-\beta_{m_{i}}m_{i}\left(t\right)-\delta_{m_{i}}m_{i}\left(t\right)ns\left(t\right)$$

(12) $$\frac{\partial ns\left(t\right)}{\partial t}=\alpha_{ns}-\beta_{ns}ns\left(t\right)-\delta_{m_{i}}m_{i}\left(t\right)ns\left(t\right)$$

Second: Coupled reaction PDE in two spatial dimensions (2D)—Model for mRNA–ncRNA interactions within a cell:

(13) $$\frac{\partial m_{i}\left(x,y,t\right)}{\partial t}=\alpha_{m_{i}}-\beta_{m_{i}}m_{i}\left(x,y,t\right)-\delta_{m_{i}}m_{i}\left(x,y,t\right)ns\left(x,y,t\right)$$

(14) $$\frac{\partial ns\left(x,y,t\right)}{\partial t}=\alpha_{ns}-\beta_{ns}ns\left(x,y,t\right)-\delta_{m_{i}}m_{i}\left(x,y,t\right)ns\left(x,y,t\right)$$

Third: Coupled reaction–diffusion PDE in two spatial dimensions (2D)—Model for mRNA–ncRNA interactions between cells:

- Intercellular ncRNA mobility
  (15) $$\frac{\partial m_{i}\left(x,y,t\right)}{\partial t}=\alpha_{m_{i}}-\beta_{m_{i}}m_{i}\left(x,y,t\right)-\delta_{m_{i}}m_{i}\left(x,y,t\right)ns\left(x,y,t\right)$$
  (16) $$\frac{\partial ns\left(x,y,t\right)}{\partial t}=d_{ns}\left[\frac{\partial^{2}ns\left(x,y,t\right)}{\partial x^{2}}+\frac{\partial^{2}ns\left(x,y,t\right)}{\partial y^{2}}\right]+\alpha_{ns}-\beta_{ns}ns\left(x,y,t\right)-\delta_{m_{i}}m_{i}\left(x,y,t\right)ns\left(x,y,t\right)$$
- Intercellular mRNA mobility
  (17) $$\frac{\partial m_{i}\left(x,y,t\right)}{\partial t}=d_{m_{i}}\left[\frac{\partial^{2}m_{i}\left(x,y,t\right)}{\partial x^{2}}+\frac{\partial^{2}m_{i}\left(x,y,t\right)}{\partial y^{2}}\right]+\alpha_{m_{i}}-\beta_{m_{i}}m_{i}\left(x,y,t\right)-\delta_{m_{i}}m_{i}\left(x,y,t\right)ns\left(x,y,t\right)$$
  (18) $$\frac{\partial ns\left(x,y,t\right)}{\partial t}=\alpha_{ns}-\beta_{ns}ns\left(x,y,t\right)-\delta_{m_{i}}m_{i}\left(x,y,t\right)ns\left(x,y,t\right)$$
- Intercellular ncRNA and mRNA mobility
  (19) $$\frac{\partial m_{i}\left(x,y,t\right)}{\partial t}=d_{m_{i}}\left[\frac{\partial^{2}m_{i}\left(x,y,t\right)}{\partial x^{2}}+\frac{\partial^{2}m_{i}\left(x,y,t\right)}{\partial y^{2}}\right]+\alpha_{m_{i}}-\beta_{m_{i}}m_{i}\left(x,y,t\right)-\delta_{m_{i}}m_{i}\left(x,y,t\right)ns\left(x,y,t\right)$$
  (20) $$\frac{\partial ns\left(x,y,t\right)}{\partial t}=d_{ns}\left[\frac{\partial^{2}ns\left(x,y,t\right)}{\partial x^{2}}+\frac{\partial^{2}ns\left(x,y,t\right)}{\partial y^{2}}\right]+\alpha_{ns}-\beta_{ns}ns\left(x,y,t\right)-\delta_{m_{i}}m_{i}\left(x,y,t\right)ns\left(x,y,t\right)$$

Fourth: Coupled reaction–diffusion PDE in multi-spatial dimensions—Model for mRNA–ncRNA interactions between cells:

- Intercellular ncRNA mobility
  (21) $$\frac{\partial m_{i}\left(x,y,\ldots ,n,t\right)}{\partial t}=\alpha_{m_{i}}-\beta_{m_{i}}m_{i}\left(x,y,\ldots ,n,t\right)-\delta_{m_{i}}m_{i}\left(x,y,\ldots ,n,t\right)ns\left(x,y,\ldots ,n,t\right)$$
  (22) $$\begin{array}{l} \frac{\partial ns\left(x,y,\ldots ,n,t\right)}{\partial t}=d_{ns}\left[\frac{\partial^{2}ns\left(x,y,\ldots ,t\right)}{\partial x^{2}}+\frac{\partial^{2}ns\left(x,y,\ldots ,n,t\right)}{\partial y^{2}}+\ldots +\frac{\partial^{2}ns\left(x,y,\ldots ,n,t\right)}{\partial n^{2}}\right]+\alpha_{ns}-\beta_{ns}ns\left(x,y,\ldots ,n,t\right)\\ -\delta_{m_{i}}m_{i}\left(x,y,\ldots ,n,t\right)ns\left(x,y,\ldots ,n,t\right) \end{array}$$
- Intercellular mRNA mobility
  (23) $$\begin{array}{l} \frac{\partial m_{i}\left(x,y,\ldots ,n,t\right)}{\partial t}=d_{m_{i}}\left[\frac{\partial^{2}m_{i}\left(x,y,\ldots ,nt\right)}{\partial x^{2}}+\frac{\partial^{2}m_{i}\left(x,y,\ldots ,n,t\right)}{\partial y^{2}}+\ldots +\frac{\partial^{2}m_{i}\left(x,y,\ldots ,n,t\right)}{\partial n^{2}}\right]+\alpha_{m_{i}}-\beta_{m_{i}}m_{i}\left(x,y,\ldots ,n,t\right)\\ -\delta_{m_{i}}m_{i}\left(x,y,\ldots ,n,t\right)ns\left(x,y,\ldots ,n,t\right) \end{array}$$
  (24) $$\frac{\partial ns\left(x,y,\ldots ,n,t\right)}{\partial t}=\alpha_{ns}-\beta_{ns}ns\left(x,y,\ldots ,n,t\right)-\delta_{m_{i}}m_{i}\left(x,y,\ldots ,n,t\right)ns\left(x,y,\ldots ,n,t\right)$$
- Intercellular ncRNA and mRNA mobility
  (25) $$\begin{array}{l} \frac{\partial m_{i}\left(x,y,\ldots ,n,t\right)}{\partial t}=d_{m_{i}}\left[\frac{\partial^{2}m_{i}\left(x,y,\ldots ,nt\right)}{\partial x^{2}}+\frac{\partial^{2}m_{i}\left(x,y,\ldots ,n,t\right)}{\partial y^{2}}+\ldots +\frac{\partial^{2}m_{i}\left(x,y,\ldots ,n,t\right)}{\partial n^{2}}\right]+\alpha_{m_{i}}\\ -\beta_{m_{i}}m_{i}\left(x,y,\ldots ,n,t\right)-\delta_{m_{i}}m_{i}\left(x,y,\ldots ,n,t\right)ns\left(x,y,\ldots ,n,t\right) \end{array}$$
  (26) $$\begin{array}{l} \frac{\partial ns\left(x,y,\ldots ,n,t\right)}{\partial t}=d_{ns}\left[\frac{\partial^{2}ns\left(x,y,\ldots ,t\right)}{\partial x^{2}}+\frac{\partial^{2}ns\left(x,y,\ldots ,n,t\right)}{\partial y^{2}}+\ldots +\frac{\partial^{2}ns\left(x,y,\ldots ,n,t\right)}{\partial n^{2}}\right]+\alpha_{ns}\\ -\beta_{ns}ns\left(x,y,\ldots ,n,t\right)-\delta_{m_{i}}m_{i}\left(x,y,\ldots ,n,t\right)ns\left(x,y,\ldots ,n,t\right) \end{array}$$

### 3.1. Two-Species Model in Dirichlet Initial Boundary Value Problem

The two-species model in two dimensions is characterized by the following system, demonstrated in Equations (27) and (28):

(27) $$\frac{\partial m_{i}\left(x,y,t\right)}{\partial t}=d_{m_{i}}\left[\frac{\partial^{2}m_{i}\left(x,y,t\right)}{\partial x^{2}}+\frac{\partial^{2}m_{i}\left(x,y,t\right)}{\partial y^{2}}\right]+\alpha_{m_{i}}-\beta_{m_{i}}m_{i}\left(x,y,t\right)-\delta_{m_{i}}m_{i}\left(x,y,t\right)ns\left(x,y,t\right)$$

(28) $$\frac{\partial ns\left(x,y,t\right)}{\partial t}=d_{ns}\left[\frac{\partial^{2}ns\left(x,y,t\right)}{\partial x^{2}}+\frac{\partial^{2}ns\left(x,y,t\right)}{\partial y^{2}}\right]+\alpha_{ns}-\beta_{ns}ns\left(x,y,t\right)-\delta_{m_{i}}m_{i}\left(x,y,t\right)ns\left(x,y,t\right)$$

The two-species model deploys the following Dirichlet boundary conditions for both the mRNA and nsRNA species:

$$\begin{array}{c} m\left(0,y,t\right)=0,\;m\left(L,y,t\right)=0\\ m\left(x,0,t\right)=0,\;m\left(x,L,t\right)=0\\ ns\left(0,y,t\right)=0,\;ns\left(L,y,t\right)=0\\ ns\left(x,0,t\right)=0,\;ns\left(x,L,t\right)=0 \end{array}$$

In this study, we present the population abundance and structure starting with a mixture of the Gaussian initial state, unless mentioned otherwise. Then, we vary the two-species model parameters to show the different patterns for population states.

### 3.2. Consistency Correction for Initial Condition and Dirichlet Boundary Conditions

The initial conditions considered in the analysis are a mixture of Gaussian states, which implies that the function at the boundaries of the initial state is not zero. However, Dirichlet-boundary conditions imply that the function at the boundaries is zero. This problem is referred to as a consistency error. The inconsistency of the initial condition and Dirichlet-boundary conditions at the boundaries are illustrated in Table 2 (0≤x,y≤L and 0≤t≤+∞).

**Figure 1:**
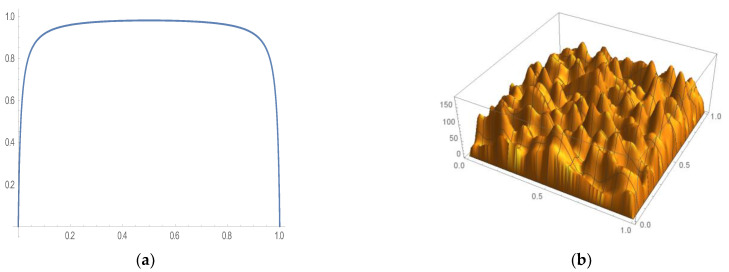
Initial condition consistency correction. (**a**) Two-dimensional initial condition consistency correction. (**b**) Three-dimensional initial condition consistency correction.

**Figure 2:**
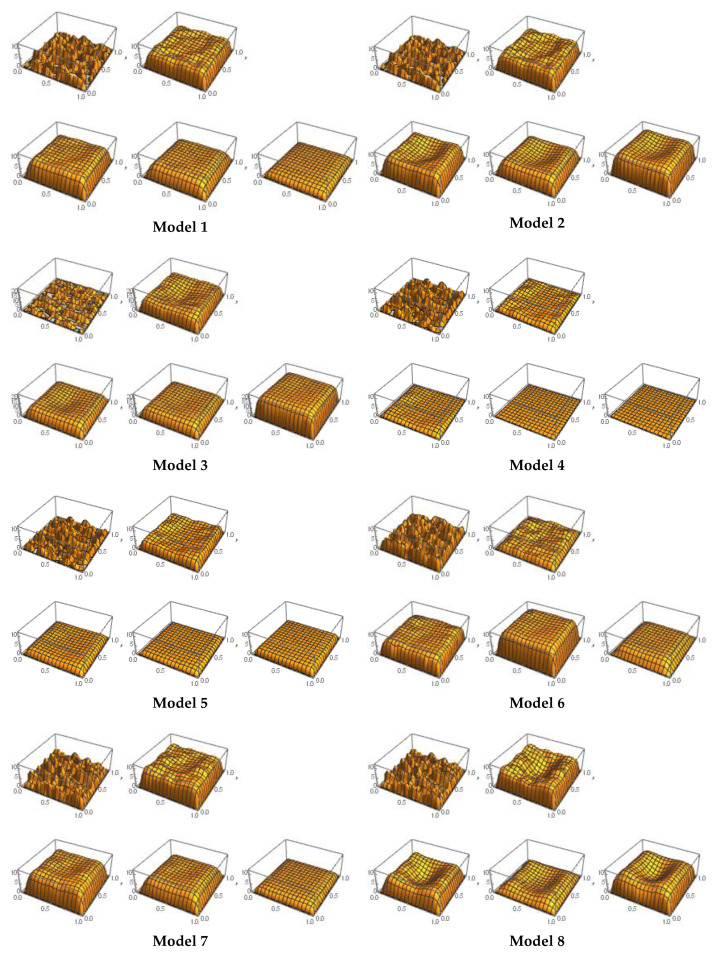

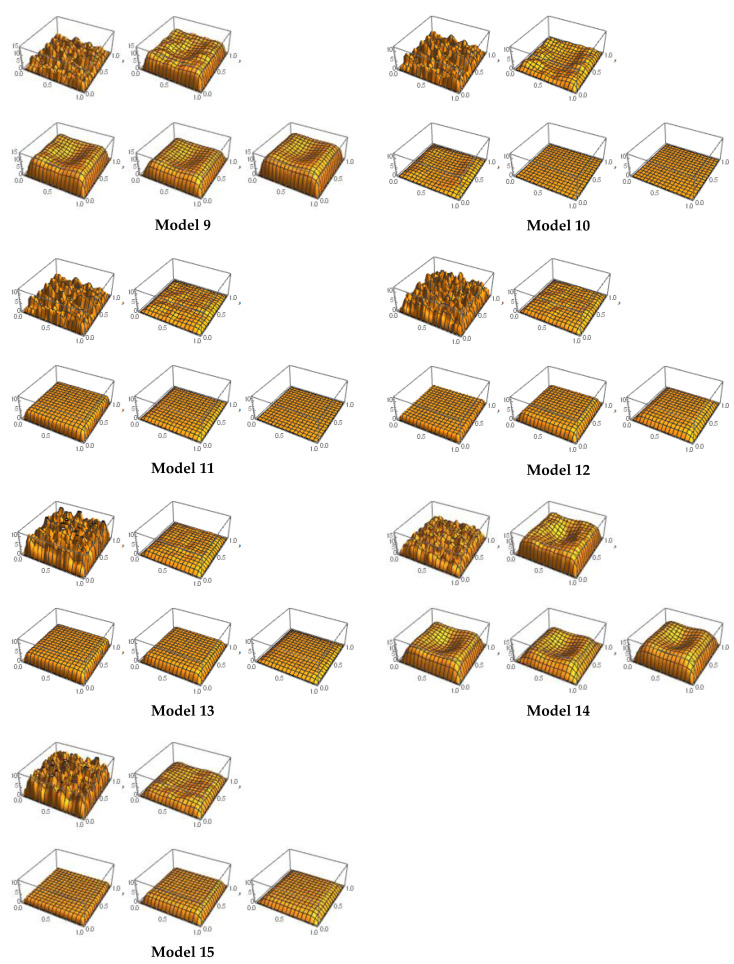
mRNA population structure at t = 0, 1, 2, 3, and 4 for each model.

The proposed research introduces a normalized-tunable sigmoid function, which upon application to the initial state levels out the boundaries to converge at zero and thus acts as a consistency correction function. The results of applying the consistency-correction function to a constant initial state, as illustrated by Equation (29), are illustrated in 2D in Figure 1 and a mixture of Gaussian initial state and 3D in Figure 2. A 1% adjustment was required in Equation (29) to ensure consistency correction as shown below.

(29) $$win\left[x\right]+win\left[y\right]=\left[\frac{-1.01x}{1-1.01-x}\times \frac{-1.01\left(1-x\right)}{1-1.01-\left(1-x\right)}\right]+\left[\frac{-1.01y}{1-1.01-y}\times \frac{-1.01\left(1-y\right)}{1-1.01-\left(1-y\right)}\right]$$

### 3.3. Two-Species Model in Neumann Initial Boundary Value Problem

The two-species model deploys the following Neumann boundary conditions for both the mRNA and nsRNA species.

$$\begin{array}{c} \frac{\partial m(0,y,t)}{\partial x}=0,\frac{\partial m(L,y,t)}{\partial x}=0,\;\frac{\partial m(x,0,t)}{\partial y}=0,\;\frac{\partial m(x,L,t)}{\partial y}=0\\ \frac{\partial ns(0,y,t)}{\partial x}=0,\frac{\partial ns(L,y,t)}{\partial x}=0,\;\frac{\partial ns(x,0,t)}{\partial y}=0,\;\frac{\partial ns(x,L,t)}{\partial y}=0 \end{array}$$

## 4. Experiments

Simulation experiments for our proposed coupled reaction–diffusion system of partial differential equations were performed using Wolfram Mathematica 11 on a computer equipped with an Intel Core i7-6600U processer, 4 CPUs, and 8GB of RAM, running Windows 10 OS.

In our previous work, we proposed a ‘fusion-based’ approach to gene expression profiling for non-small cell lung cancer [12]. The proposed approach was based on three main steps: (1) clustering tendency to determine whether the considered data could be grouped into clusters; (2) evidence-based fusion to combine the clustering results in the three non-treatment (NT) samples; and (3) ‘fuzzy c-means-with-range clustering’ performed on the short-exposure (SE) and long-exposure (LE) samples. The main goal of this last step was to perform clustering on the SE and LE data while also taking into account the range of cluster sizes previously obtained in earlier steps. Finally, we explored the presence of change in cluster size among all three conditions: NT, SE, and LE. A description of these three conditions is depicted in Table 3. This work helped us to identify genes that migrate from one cluster to another. Hence, we can evaluate the effect that non-thermal plasma treatment may have on tumors by determining the change in membership for each individual gene. In the current paper, we use the results of clustering made on the same dataset to validate our proposed approach.

In this section, we begin by describing the dataset used for the validation of the proposed model. Then, we detail computational results displaying the two-species interactions in a two-dimensional environment.

### 4.1. Dataset

Plasma, a state of matter with gas-like properties and low density, is usually created by ionization, a process in which the atoms or molecules of a gas acquire a stronger charge, either positive or negative, through the application of high heat or subjection to a strong electromagnetic field at similarly high temperatures. Plasma may be either thermal or non-thermal, with thermal plasma having a single stable temperature across its electrons, ions, and neutral particles, whereas non-thermal plasma is characterized by having electrons with higher temperatures than its ions and neutral particles [40]. Recent advances in medical technology have made the utilization of plasma a reality for medical treatment in various capacities, and cancer is one of the many diseases upon which the efficacy of new plasma-based treatments is being tested.

One of the datasets from previous iterations of this research provides the data we work from here. Hou et al. [41] provide the gene-expression profile of the tumor cells of lung adenocarcinoma following treatment using non-thermal atmospheric plasma at various times and rates of exposure. The data gathered by Hou et al. in this process include the original transcriptome of the tumor cell, the state of the transcriptome at the moment of short-exposure plasma treatment, and finally, the transcriptome following long-exposure plasma treatment. All data gathered by Hou et al. is publicly available through the NCBI Gene Expression Omnibus, and no permissions were required (accessible at #GSE59997).

### 4.2. Experimental Results

In this section, we present computational results displaying the two-species interactions in a two-dimensional environment. The two species used are messenger RNA (mRNA) and small interfering RNA (nsRNA). For all of the following numerical simulations, we set $D_{m_{i}}=D_{ns}=10^{-3}$ and Ω=[0,1]. Parameters for both mRNA and nsRNA; transcription rate, self-degradation rate, and coupled degradation were chosen based on experimental rates as mentioned by [42,43,44]. These works also introduced the epigenetic rate, in addition to the logarithmic and exponential rates, to examine the sensitivity of parameters tuning to the mRNA population state. In the present work, we refer to the population state as not only the population abundance but also the population structure.

Parameters displayed in Table 4 were used in the two-species system of partial differential equations. The basis for these equations is the biological interpretation of RNA transcription and degradation, which is assumed to rely on exponential constructs.

Table 5 displays the mathematical formula for the parameters of each model, from 1 to 15. It presents the transcription, self-degradation, and coupled-degradation functions for both species: mRNA and nsRNA.

**Figure 3:**
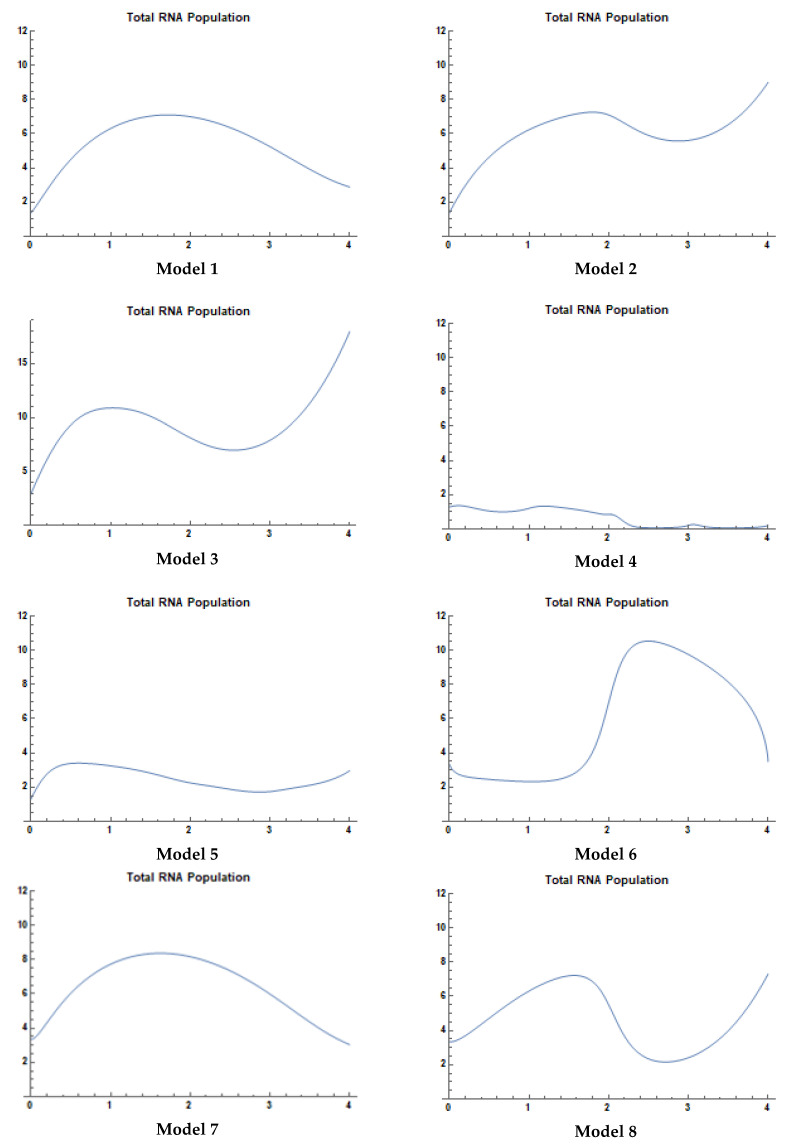

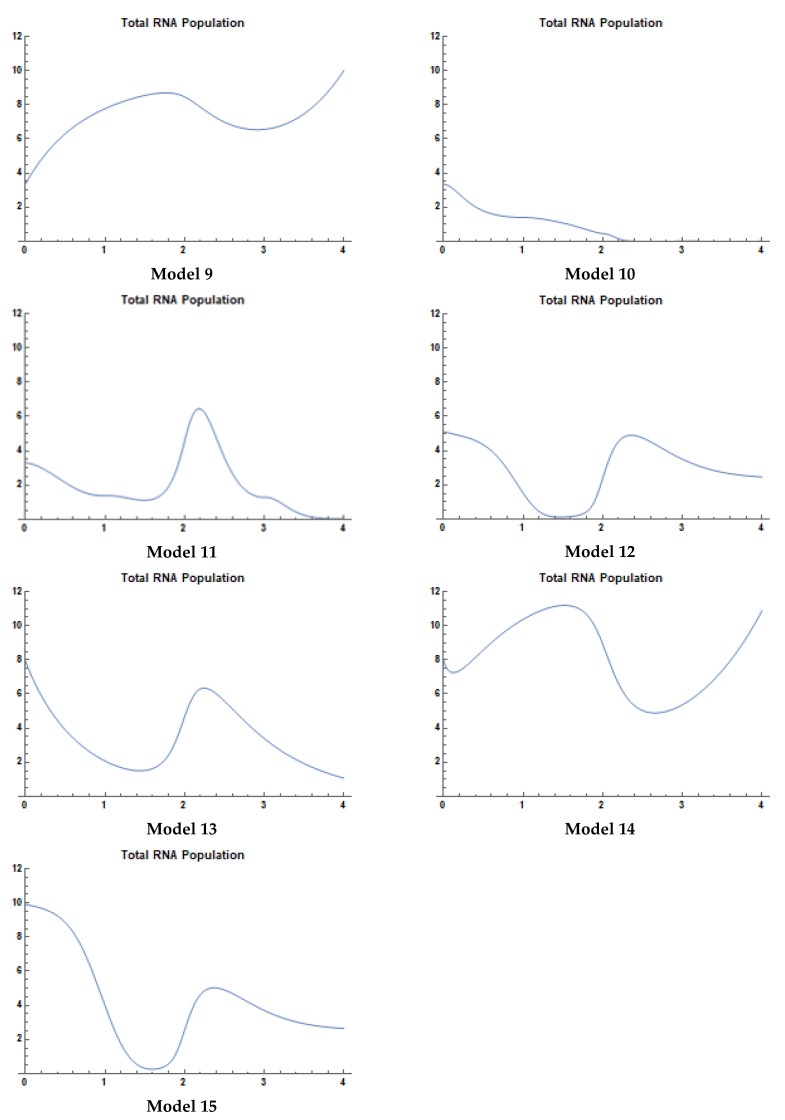
Total mRNA population abundance at t = 0, 1, 2, 3, and 4 for each model.

Figure 2 and Figure 3 present the spatiotemporal variation in the mRNA population. Figure 2 shows the mRNA population structure for each of the 15 models, as a mixture of Gaussian distribution at the initial state (t = 0) and the composition of this structure through t = 1, 2, 3, and 4. The initial state shows a heterogeneous Gaussian construct from a temporal spatial perspective, and following the application of the models, spatial changes are homogenized based on transitioning from time t = 1 to time = 4. The capability to model tumorigenesis from the two-species construct is thereby enhanced, and leads to improved gene expression profiling.

However, Figure 3 displays the variation in total mRNA population abundance at different transition time states (t = 0, 1, 2, 3, and 4) for each of the 15 models. Areas with troughs in mRNA abundance limit the model’s ability to garner biomarkers from the dataset and negatively impact the efficacy of tumor biomarker profiling.

The population abundance or concentration of mRNA in Figure 3 was discretized to four clusters based on the optimal numbers of clusters obtained from our previous work [12]. The scaling of the four clusters is displayed in Table 6, which depicts the lower and upper bound for each cluster (the clustering boundaries). Hence, each of the mRNA–nsRNA interaction models presents a distinct pattern of clustering membership, from t = 0 to t = 4.

In Table 7, we present the molecular expression profiling that depicts biological biomarkers for non-small lung cancer following the fusion of various samples: NT samples, LE post 1 h samples, LE post 2 h samples, and LE post 4 h samples. The biomarkers that could potentially signify tumorigenesis and lung cancer were clustered according to our previous clustering results, and include AKT1, ALK, BRAF, EGFR, HER2, KRAS, MEK1, MET, NRAS, PIK3CA, RET, and ROS1 [12]. The oncogenes BRAF, RET, ROS1, MET, ALK, and PIK3CA each reveal a transition of overexpression from the NT state to the non-thermal plasma treatment states, whereas SMEK1 is the only gene exhibiting a suppression during movement from the NT state to the LE states. Despite these differences, all genes demonstrate consistency in gene expression throughout the non-thermal plasma treatment at all three hourly intervals: post 1 h, post 2 h, and post 4 h.

Our paper introduces a mathematical model that allows for the profiling of gene-expression dynamics and connects these using a fusion clustering approach based on qualitative evidence. The last column in Table 7 presents the mRNA–nsRNA interaction model that is either the best or most complete fit for the clustering pattern of each oncogene. Model matching was performed by comparing gene-expression patterns prior to and after modelling (in Wolfram Mathematica), with the outcomes summarized below:

- Model 1 is the best fit for MET1, KRAS1, NRAS2, EGFR1, and PIK3CA3 patterns.
- Model 2 is the complete fit for MET4 pattern.
- Model 3 is the complete fit for MET2 pattern.
- Model 4 is the complete fit for AKT2, EGFR7, and PIK3CA2 patterns.
- Model 5 is the best fit for KRAS3, EGFR4, and BRAF patterns.
- Model 6 provides the best fit for RET1 patterns.
- Model 7 is the complete fit for ALK2 pattern.
- Model 8 is the best fit for AKT3, EGFR5, RET2, and RET3 patterns.
- Model 9 is the complete fit for ALK1 pattern.
- Model 10 is the complete fit for EGFR8 pattern.
- Model 11 is the best fit for ROS1 patterns.
- Model 12 is the best fit for EGFR6 patterns.
- Model 13 is the best fit for AKT1, NRAS1, and EGFR3 patterns.
- Model 14 is the complete fit for MET3, KRAS4, and EGFR2 patterns.
- Model 15 is the complete fit for SMEK1 pattern.

## 5. Conclusions

In this paper, we present a novel mathematical reaction–diffusion system as an alternative approach for explainable gene expression profiling. Our study points to several observations that can be gleaned from the quantitative/qualitative analysis applied to mRNA–ncRNA interactions. First, the rate of transcription in mRNA, the rate of self-degradation in mRNA, and the rate of transcription in siRNA are all effective components of the mRNA partial differential equation. Second, the rate of self-degradation in siRNA and the rate of transport in siRNA within a tissue are effective components in the siRNA partial differential equation. Additionally, the coupled-degradation rate of mRNA and siRNA is an effective component in the coupled system of partial differential equations. Finally, the transport of mRNA within a tissue is an effective part of the mRNA partial differential equations.

Our current work can be further extended by considering dynamic relationships among genes. Such relationships can reveal cascade effects and interactions among genes, indicating that the expression of one particular gene may alter the rate of transcription of another. In addition, dynamic relationships may reveal patterns representing genes of similar expressions (i.e., co-regulating one another or else regulated by a parent gene). Hence, modelling a system of partial differential equations in which simultaneous genes are considered could result in an effective gene regulatory system. For future work, our novel reaction diffusion system for gene expression profiling could be utilized in the context of SARS-CoV-2-infected human cell lines to identify molecular targets for therapeutic intervention [45]. Building on the approach of Bar-Sinai et al. [46], alternative interpretable approaches can be explored in the future by applying ML models to partial differential equations to recover unknown dynamics in gene regulatory systems. The role of advanced biosensor techniques for gene expression profiling as part of ML models could also be considered in this context [47,48].

## Figures and Tables

**Table 1:** List of mathematical notations.

Notation	Description
$m_{i}$	Messenger RNA (mRNA) Population
ns	Non-coding small interfering RNA (siRNA) Population
$\alpha_{m_{i}}$	Production rate of mRNA
$\beta_{m_{i}}$	Self-degradation rate of mRNA
$\delta_{m_{i}}$	Coupled Degradation rate of mRNA due to siRNA
$\alpha_{ns}$	Production rate of siRNA
$\beta_{ns}$	Self-degradation rate of siRNA
$D_{m_{i}}$	Diffusion coefficient of mRNA
$D_{ns}$	Diffusion coefficient of siRNA

**Table 2:** Boundaries at the initial condition (IC) and Dirichlet boundary conditions.

Boundaries of IC	Dirichlet B.Cs
m(0,y,0)≠0 m(L,y,0)≠0 m(x,0,0)≠0 m(x,L,0)≠0	m(0,y,t)≠0 m(L,y,0)≠0 m(x,0,t)≠0 m(x,L,t)≠0

**Table 3:** Dataset notations.

NT	Non-Treatment or Control Group
SE	Short-exposure non-thermal plasma treatment, measured post 1 h of treatment
LE post 1 h	Long-exposure non-thermal plasma treatment, measured post 1 h of treatment
LE post 2 h	Long-exposure non-thermal plasma treatment, measured post 2 h of treatment
LE post 4 h	Long-exposure non-thermal plasma treatment, measured post 4 h of treatment

**Table 4:** Transcription, self-degradation, and coupled degradation functions.

Constant	Exponential	Log
$10^{-3}$ $10^{-2}$ $10^{-1}$ 1 10 $10^{2}$ $10^{3}$	$e^{t}$ $e^{-10^{2}{\left(t-2\right)}^{2}}$ $e^{-{\left(t-2\right)}^{2}}$ $e^{3-t}+e^{-1+t}$ $0.5(e^{3-t}+e^{-1+t})$ $\frac{e^{-{\left(t-2\right)}^{2}}}{0.05+{\left(t-2\right)}^{2}}$ $e^{\frac{t}{2}}\left|\sin\left(\pi t\right)\right|$	log(1+t) $\frac{1}{\log\left(5\right)-\log\left(1+t\right)}$
		Epigenetic
		$1+\frac{1}{2-10^{-1}\left(t\right)}$
		Experimental
		$0.5\left[Tanh\left(\frac{xy-0.5}{0.2}\right)+1\right]$ $Tanh\left(\frac{xy-0.5}{0.2}\right)+1$

**Table 5:** Transcription, self-degradation, and coupled degradation functions for each model.

Model	$\alpha_{m}$	$\beta_{m}$	$\alpha_{ns}$	$\beta_{ns}$	$\theta_{m}$
1	$0.5(e^{3-t}+e^{-1+t}$)	$10^{-3}$	$e^{t}$	10	1
2	$0.5(e^{3-t}+e^{-1+t}$)	$10^{-3}$	$\frac{e^{-{\left(t-2\right)}^{2}}}{0.05+{\left(t-2\right)}^{2}}$	$10^{-1}$	$10^{-1}$
3	$e^{3-t}+e^{-1+t}$	$e^{-{\left(t-2\right)}^{2}}$	$0.5\left[Tanh\left(\frac{xy-0.5}{0.2}\right)+1\right]$	$10^{-2}$	$e^{-{\left(t-2\right)}^{2}}$
4	$0.5\left[Tanh\left(\frac{xy-0.5}{0.2}\right)+1\right]$	$e^{-10^{2}{\left(t-2\right)}^{2}}$	$\frac{e^{-{\left(t-2\right)}^{2}}}{0.05+{\left(t-2\right)}^{2}}$	$10^{-2}$	$e^{\frac{t}{2}}\left|\sin\left(\pi t\right)\right|$
5	$0.5(e^{3-t}+e^{-1+t}$)	log(1+t)	$e^{\frac{t}{2}}\left|\sin\left(\pi t\right)\right|$	$e^{\frac{t}{2}}\left|\sin\left(\pi t\right)\right|$	log(1+t)
6	$\frac{e^{-{\left(t-2\right)}^{2}}}{0.05+{\left(t-2\right)}^{2}}$	$10^{-2}$	$\frac{1}{\log\left(5\right)-\log\left(1+t\right)}$	10	1
7	$0.5(e^{3-t}+e^{-1+t}$)	$10^{-3}$	$e^{t}$	10	1
8	$0.5(e^{3-t}+e^{-1+t}$)	$10^{-3}$	$\frac{e^{-{\left(t-2\right)}^{2}}}{0.05+{\left(t-2\right)}^{2}}$	$10^{-3}$	1
9	$0.5(e^{3-t}+e^{-1+t}$)	$10^{-3}$	$\frac{e^{-{\left(t-2\right)}^{2}}}{0.05+{\left(t-2\right)}^{2}}$	$10^{-1}$	$10^{-1}$
10	$0.5\left[Tanh\left(\frac{xy-0.5}{0.2}\right)+1\right]$	$e^{-10^{2}{\left(t-2\right)}^{2}}$	$\frac{e^{-{\left(t-2\right)}^{2}}}{0.05+{\left(t-2\right)}^{2}}$	$10^{-2}$	$e^{\frac{t}{2}}\left|\sin\left(\pi t\right)\right|$
11	$\frac{e^{-{\left(t-2\right)}^{2}}}{0.05+{\left(t-2\right)}^{2}}$	$e^{\frac{t}{2}}\left|\sin\left(\pi t\right)\right|$	$e^{\frac{t}{2}}\left|\sin\left(\pi t\right)\right|$	$e^{-{\left(t-2\right)}^{2}}$	$10^{-3}$
12	$\frac{e^{-{\left(t-2\right)}^{2}}}{0.05+{\left(t-2\right)}^{2}}$	$10^{-2}$	$e^{3-t}+e^{-1+t}$	$\frac{e^{-{\left(t-2\right)}^{2}}}{0.05+{\left(t-2\right)}^{2}}$	$\frac{e^{-{\left(t-2\right)}^{2}}}{0.05+{\left(t-2\right)}^{2}}$
13	$\frac{e^{-{\left(t-2\right)}^{2}}}{0.05+{\left(t-2\right)}^{2}}$	1	$0.5\left[Tanh\left(\frac{xy-0.5}{0.2}\right)+1\right]$	$10^{-3}$	$10^{-1}$
14	$0.5(e^{3-t}+e^{-1+t}$)	$10^{-3}$	$\frac{e^{-{\left(t-2\right)}^{2}}}{0.05+{\left(t-2\right)}^{2}}$	$10^{-3}$	1
15	$\frac{e^{-{\left(t-2\right)}^{2}}}{0.05+{\left(t-2\right)}^{2}}$	$10^{-2}$	$e^{3-t}+e^{-1+t}$	$\frac{e^{-{\left(t-2\right)}^{2}}}{0.05+{\left(t-2\right)}^{2}}$	$\frac{e^{-{\left(t-2\right)}^{2}}}{0.05+{\left(t-2\right)}^{2}}$

**Table 6:** Normalized scale of population abundance.

	Normalized Scale	
**C1**	0	2.265458
**C2**	2.267602	4.383277
**C3**	4.383667	7.001413
**C4**	7.002388	12

**Table 7:** Molecular profiling of non-small lung cancer (NSLC) oncogenes.

	Fusion NT	Fusion LE Post 1 h	Fusion LE Post 2 h	Fusion LE Post 4 h	Model
***AKT1(1)***	*4*	*1*	*1*	*1*	*13*
***AKT1(2)***	*1*	*1*	*1*	*1*	*4*
***AKT1(3)***	*2*	*3*	*3*	*3*	*8*
***MET(1)***	*1*	*3*	*3*	*3*	*1*
***MET(2)***	*1*	*4*	*4*	*4*	*3*
***MET(3)***	*4*	*4*	*4*	*4*	*14*
***MET(4)***	*1*	*3*	*4*	*4*	*2*
***KRAS(1)***	*1*	*3*	*3*	*3*	*1*
***KRAS(2)***	*4*	*1*	*1*	*2*	*NA*
***KRAS(3)***	*1*	*2*	*2*	*2*	*5*
***KRAS(4)***	*4*	*4*	*4*	*4*	*14*
***NRAS(1)***	*4*	*1*	*1*	*1*	*13*
***NRAS(2)***	*1*	*3*	*3*	*3*	*1*
***EGFR(1)***	*1*	*3*	*3*	*3*	*1*
***EGFR(2)***	*4*	*4*	*4*	*4*	*14*
***EGFR(3)***	*4*	*1*	*1*	*1*	*13*
***EGFR(4)***	*1*	*2*	*2*	*2*	*5*
***EGFR(5)***	*2*	*3*	*3*	*3*	*8*
***EGFR(6)***	*3*	*2*	*2*	*2*	*12*
***EGFR(7)***	*1*	*1*	*1*	*1*	*4*
***EGFR(8)***	*2*	*1*	*1*	*1*	*10*
***BRAF***	*1*	*2*	*2*	*2*	*5*
***ALK(1)***	*2*	*4*	*4*	*4*	*9*
***ALK(2)***	*2*	*4*	*4*	*2*	*7*
***PIK3CA(1)***	*1*	*3*	*3*	*3*	*1*
***PIK3CA(2)***	*1*	*1*	*1*	*1*	*4*
***SMEK1***	*4*	*2*	*2*	*2*	*15*
***RET(1)***	*2*	*2*	*2*	*2*	*6*
***RET(2)***	*2*	*3*	*3*	*3*	*8*
***RET(3)***	*2*	*3*	*3*	*3*	*8*
***ROS1***	*2*	*3*	*3*	*1*	*11*

## Data Availability

The data used in this paper was gathered by Hou et al. [41] and is publicly available through the NCBI Gene Expression Omnibus. No permissions were required (accessible at #GSE59997).
